# Establishing Clinical Ethics Committees in Primary Care: A Study from Norwegian Municipal Care

**DOI:** 10.1007/s10730-021-09461-9

**Published:** 2021-10-01

**Authors:** Morten Magelssen, Heidi Karlsen, Lisbeth Thoresen

**Affiliations:** 1 Centre for Medical Ethics, Institute of Health and Society, University of Oslo, Oslo, Norway; 2 Department for Interdisciplinary Health Sciences, Institute of Health and Society , University of Oslo, Oslo, Norway

**Keywords:** Clinical ethics, Clinical ethics committee, Clinical ethics support, Community care, Home care, Municipal care, Nursing home, Primary care

## Abstract

Would primary care services benefit from the aid of a clinical ethics committee (CEC)? The implementation of CECs in primary care in four Norwegian municipalities was supported and their activities followed for 2.5 years. In this study, the CECs’ structure and activities are described, with special emphasis on what characterizes the cases they have discussed. In total, the four CECs discussed 54 cases from primary care services, with the four most common topics being patient autonomy, competence and coercion; professionalism; cooperation and disagreement with next of kin; and priority setting, resource use and quality. Nursing homes and home care were the primary care services most often involved. Next of kin were present in 10 case deliberations, whereas patients were never present. The investigation indicates that it might be feasible for new CECs to attain a high level of activity including case deliberations within the time frame. It also confirms that significant, characteristic and complex moral problems arise in primary care services.

## Introduction

Would primary care services benefit from the aid of a clinical ethics committee (CEC)? While CECs have been established in hospitals in many Western countries, there is little research on CECs in primary care (Doran et al., 2016; Slowther et al., 2004). By “primary care” and “community care” we refer to non-hospital health and social care services provided locally in the community. Our group has supported the implementation of four CECs in Norwegian primary care, and in the present article we report on the status of the CECs 2.5 years after their planned establishment.

### Research on CECs in Primary Care

Recent reviews present the literature on CECs and other ethics support in nursing homes/residential long-term care (Holmes et al., 2020; van der Dam et al., 2014). A 2020 review on CECs in this setting found only 13 primary studies, of which four were from Europe and the remainder from the US (Holmes et al., 2020). None of these were rigorous evaluation studies. However, CECs that serve nursing homes apparently typically do not serve *other* services within primary care. Outside of Norway we have not found reports about CECs that serve a municipality or community’s primary health and (social) care services in full.

### The Norwegian Context

Societies organize primary care differently. In Norway (pop. 5.3 million), health and social care is mostly publically financed, and primary care is organized by the 356 municipalities. Primary care includes services such as nursing homes, home-based care, sheltered housing/assisted living facilities, school health services, child and adolescent health centres, substance abuse and mental health services, emergency rooms, and general practitioners.

### The Project

The present project involves the implementation of CECs in four Norwegian municipalities. The project has been presented in detail in a separate article (Magelssen et al., 2020a). The municipalities each appointed two “resource persons” who would then assume main responsibility for establishing the CEC and become its leader and secretary. Researchers from the Centre for Medical Ethics (CME) at the University of Oslo provided training and implementation support before and throughout the study period which ran from Jan 2018 [= M1 (month 1)] to Jun 2020 (= M30). The four municipalities were located in the South-East region of Norway and had populations ranging from just over 10,000 (CEC 4) to more than 80,000 (CEC 1). Whereas CECs 1–3 were intended to serve the full range of the municipality’s health and care services, CEC 4 was to serve the entire municipality, in other words, also municipal services outside of the health and care sector.

### Aim

The aim of the present article is to chart the structure and activities of the CECs 2.5 years after their establishment, with special emphasis on what characterizes the cases they have discussed.

## Methods

### Data Sources

The data used in the present article stem from three sources: First, the four CECs wrote yearly reports for 2018 and 2019. These are publically available. Second, the CECs contributed anonymized case reports from each case deliberation. Third, for each case deliberation the CEC would also fill a brief form with information about the deliberation, such as who was present, the duration of the deliberation, and a simple self-evaluation. The yearly reports and the case reports were written as an integral part of the CECs’ work, not specifically for this research project. The CEC’s resource persons were contacted at the end of the study period to clarify or provide any missing information.

### Analysis

Case reports were analyzed in order to understand and characterize the ethical issues involved in the case. CECs were encouraged to formulate the main ethical issues with precision, as a key part of the deliberation process. The CEC’s own formulation of the ethical issues was validated, supplemented and sometimes adjusted through our reading of the full case report. Then, main categories were formed. MM and HK read all case reports and all three authors discussed in order to achieve agreement on interpretations and categorization. The analysis and categorization of the ethical issues involved was based on the case reports and thus inductive but was also a hermeneutic process involving our preconceptions of what an ethical issue is, what kind of issues typically arise in primary care, how CECs are supposed to work, and of how similar studies of hospital CECs have categorized ethics issues. We define an ethical issue in patient care as a situation in which there is doubt, uncertainty or disagreement about what is good or bad, or right or wrong to do. The self-evaluations were analyzed qualitatively.

### Research Ethics

The study was evaluated by the Data Protection Official at the Norwegian Centre for Research Data (project no. 56714). According to the Norwegian system, no further ethics approval was required. CEC members gave informed consent to participate in the study. Case reports were anonymized and thus did not identify patients, next of kin or professionals.

## Results

### The Committees

CECs 1&2 were in operation from M1, CEC 3 from M2, whereas CEC 4 began in M17. In all four municipalities the CECs were placed high in the organizational hierarchy: either directly under the leader for the health and care services (CECs 2&3), in the section for health and welfare (CEC 1), or directly under the municipal commissioner who leads the municipal bureaucracy (CEC 4).

At M24, the number of members of each CEC ranged from seven (CEC 2) to 13 (CEC 4). All committees had at least one physician, nurse, and lay member. Other health professions represented were nurses’ aides (2 CECs), social worker (2), psychologist (1), school health nurse (1), and physiotherapist (1). Two CECs had a municipal lawyer, whereas one CEC invited a lawyer when required. One CEC had an academic ethicist. Clergy were members of three CECs, whereas two CECs had municipal mid-level managers as members. Two CECs were led by physicians, one by a physiotherapist and one by a palliative nurse with responsibility for coordination of palliative services.

### Characteristics of Case Deliberations and Ethical Issues Involved

The four CECs discussed 54 cases in total in the study period (CEC 1&2: 19 each; CEC 3: 10; CEC 4: 6). CEC 1 received the bulk of their cases (12 of 19) in year 2; CEC 2 in year 1 (10 of 19). Of the 54 cases, 39 were prospective cases concerning individual patients. A further 4 cases were retrospective, whereas 11 cases concerned general or principled issues (i.e., not connected to specific individuals). Table 1 summarizes the main ethical issues discussed.

**Table 1:** Main ethical issues in 51 CEC case deliberations

Ethical issue	# of cases	Examples
Patient autonomy, competence and coercion	20	Should the patient be admitted to a nursing home against their will? Should the patient with dementia be restricted in taking unsupervised walks, due to safety? (see Magelssen & Karlsen, 2021)
Professionalism	10	Should home care nurses refuse to provide care because the patient’s home provides a poor working environment? How far should the professional "stretch" themselves in providing services?
Cooperation and disagreement with next of kin	9	Is it acceptable that next of kin forcibly feed the patient who has a short life-expectancy? What should professionals do when the patient’s legal guardian limits the patient’s freedom unreasonably?
Priority setting, resource use and quality	8	What is the proper level and site of care for a young patient with a history of substance abuse, currently residing in a nursing home? How should the views and needs of the patient’s spouse be weighed in the question of level of care and resource use?
Decision-making and care at the end of life	6	Should artificial nutrition and hydration be instigated for a patient without competence to consent who has stopped eating and drinking? How should professionals handle disagreement with next of kin about care towards the end of life?
Interests of the one vs. interests of the many	6	How should the nursing home weigh infection control and the need for patients to receive visits? How should interests be balanced when coercive measures for some patients influence other patients on the ward negatively?
Information and confidentiality	2	Should a spouse’s wish that the patient not be informed about a progressive, ultimately lethal condition be respected?
Encounters between cultures	1	Can next of kin demand that the patient not be cared for by a professional of the opposite sex?

Three of the 54 cases, originating from CEC 4, did not concern the health and care sector; these cases have not been categorized. Several cases have been placed in two categories

#### Patient Autonomy, Competence and Coercion

In a large subset of cases, issues of patient autonomy, competence and coercion were central—and intertwined. Patients typically either lacked competence to consent or their competence was put in question; many had a diagnosis of dementia or serious chronic mental illness. The use of coercion then often became a potential line of action if patient preferences collided with staff’s perception of the patient’s best interests. In some cases, it was asked whether the patient who refuses help has "a right to perish" or whether professionals should arrange for, e.g., forced admission to a nursing home. In other cases, there was a question of when, how and to what extent professionals might reasonably set limits to patients’ freedom—when it comes to freedom of movement, sexual relations, and smoking or potentially dangerous activities. Thus, autonomy and freedom versus safety and security was a recurring value conflict. Another issue was coercion in personal hygiene; yet another issue was how to handle that some patients might be indirectly affected by coercive measures for other patients.

#### Professionalism

Issues of professionalism and the role and responsibilities of the professional were brought up in several cases. Some cases concerned moral distress and how far the professional should go to exercise good care. For instance, several cases from home care concerned difficult or unacceptable working environments—physically, or psychologically due, e.g., to threats and verbal abuse. How much should professionals be expected to tolerate? Would they be justified in limiting or refraining from providing care altogether? Professionalism was also addressed in a case concerning whether professionals might justifiably develop friendly relations with patients, and in a case about responsibility to minimize risk of Covid-19 infection in refraining from activities in one’s spare time. Some cases also concerned alleged insufficient competence and quality of care among colleagues and what to do about it.

#### Cooperation and Disagreement with Next of Kin

In several cases, next of kin (or legal guardians) played a central role. Sometimes professionals and next of kin disagreed on decisions about care, such as the medications or level of care the patient should receive. Sometimes cooperation was difficult. Some professionals observed violent or otherwise harmful or unhealthy relations between patients and next of kin and wondered whether and how they could intervene. As next of kin played important roles as informal carers, professionals would sometimes need guidance on setting limits to next of kin’s involvement in care.

#### Priority Setting, Resource Use and Quality

Some cases addressed fair use of resources and concerns about quality of care. Cases could involve uncertainty about proper level of services; for instance, should the patient be admitted to a nursing home? Ought dying patients to have a member of staff allocated to them at all times? In some cases, there was disagreement between different services in the municipality or between municipal services and the local hospital. Whether and how next of kin’s needs and preferences should be taken into account in resource allocation was also discussed. Furthermore, priority setting and resource use was brought up as an additional element in several other cases where other issues took centre stage.

#### Decision-Making and Care at the End of Life

Some cases concerned the level of treatment to provide at the last stages of life. How should such decisions be made for a patient who refuses to participate in decision-making? Should artificial nutrition and hydration be instigated for a patient without competence to consent who has stopped eating and drinking? Several cases involved disagreement with next kin who for instance would insist on artificial nutrition or claim that the patient received too aggressive pain medication.

#### Interests of the One vs. Interests of the Many

The Covid-19 pandemic raised issues of infection control where the interests of the one must be balanced with the interests of others. For instance, restrictions on visits and freedom of movement became important issues to address in nursing homes and sheltered housing where patients live together. However, the topic was also relevant outside of the context of the pandemic. Coercive measures for some patients could lead to negative effects for other patients on the ward; how were interests to be balanced? When patients were violent or acted out, their interests must be balanced with the safety of other patients and staff.

#### Information and Confidentiality; Encounters Between Cultures

In two cases professionals and next of kin disagreed about how much information should be given to patients lacking competence to consent. A further case concerned disagreement about care being provided to a patient by a carer of the opposite sex, something which next of kin found unacceptable. Here, cultural differences were found to play a role in the conflict.

#### Professional, Practical or Ethical Issues?

Most cases discussed in the CECs were complex, and from the case reports we see how the CECs typically has taken care to identify issues that are genuinely ethical from ‘within’ the full situation as presented to them. In the most complex cases, professional, practical and ethical issues were clearly intertwined; in five cases of this kind an ethical issue was not sharply demarcated or formulated in the case report.

### How Case Deliberations Took Place

For the 50 case deliberations where we have sufficient information, the full CEC participated in 36, whereas in 14 cases two to four CEC members were present. There were no instances of consultations performed by a single CEC member only.

The CECs produced a full-text case report in 30 cases, whereas they wrote a simplified report with keywords in 23 cases. The latter format was especially practiced by CEC 2 (13 of 19 cases). In one case, no report was written.

We have complete or partial data from the CECs’ self-evaluation reports in 33 of 54 cases. The general impression is that the CECs typically were satisfied with how the case consultations went, with the highest self-assessment scores towards the end of the study period. When stakeholders (i.e., staff and next of kin) were present, the CECs rated highly the way they included and cared for them. The CECs reported that they sometimes lacked competence required in a case, and then most often specified this as knowledge of relevant law. Case deliberations lasted on average 81 minutes (range 50–120).

### Professionals and Services Involved in Case Deliberations

Notably, many cases were referred from mid-level managers or from CEC members; relatively few originated from staff directly involved in patient care (Table 2). Not a single case was referred from physicians who were not themselves CEC members.

**Table 2:** Who referred cases to the CEC? 40 of 54 cases accounted for

Profession/role	# of cases
*Leaders at different levels*	
Mid-level managers (wards, units)	16
Municipal top-level manager	2
*Staff directly involved in patient care*	
Nurse	4
Health professional with coordinator role	4
Physiotherapist	2
Occupational therapist	1
Learning disability nurse	1
*Others*	
CEC members	7^a^
Municipal office staff	3

^a^Most common at the start of the study period

Half of all cases involved nursing homes, whereas many also involved home care services (Table 3). Several important municipal services were not involved in any of the CEC cases. These services include school health services, general practitioners, emergency rooms, and child and adolescent health centres.

**Table 3:** Services involved in the cases discussed in the CECs. 50 of 54 cases accounted for

Service	# of cases
Nursing home	27
Home care	11
Sheltered housing/services for the disabled	6
Services outside of health and care	4
Rehabilitation/lifestyle and coping services	3
Mental health and substance abuse care	2
Municipal service allocation office	2

More than one service was involved in five cases

Professionals from the relevant department(s) participated in 43 case deliberations, whereas in five they were not invited. For the remaining six deliberations we lack information. Next of kin took part in 10 case deliberations (5 each in CEC 1&2). In one further case, they declined participation. In a further 27 cases where next of kin were involved, they were not invited. However, case reports show that in several of the cases the CEC has suggested that next of kin be invited, but staff members with whom the case originated have not wanted this. Patients were not present at any case deliberation.

### Other Activities

In addition to case consultations, the CECs also undertook other activities, examples of which are given in Table 4.

**Table 4:** Examples of further activities undertaken by the CECs in the study period

	CEC 1	CEC 2	CEC 3	CEC 4
Presentations for municipal managers	Multiple each year	Yearly	Multiple each year	Twice, including municipal council
Visits to services	Ethics training for nursing home staff	Ethics training for nurses’ aides in further education		
Seminars for staff	On ethics reflection and priority setting for 330 staff and managers	On autonomy and competence for 70 staff	On ethics reflection for > 100 staff	
Contributions to guideline development		On visitation rules during covid pandemic	On visitation rules during covid pandemic	
CEC meetings in total in study period	26	47	21	7

## Discussion

### The Nature of Ethical Issues in Municipal Care

The cases provide an interesting window into the ethical challenges faced by primary care services. Of course, a CEC can only expect to see “the tip of the iceberg” of the ethical issues that arise in the services. Yet, the cases clearly indicate that primary care is faced with significant and complicated value conflicts, the successful handling or resolution of which will make a difference—ultimately also for patients and the services they receive, and for next of kin. Patient autonomy, competence and coercion in particular loom large. Many service recipients in primary care are elderly and frail and the question of competence to consent arises. It is not surprising that this leads to moral challenges. These challenges are often complex, not least because of the complex nature of the services and their interplay, and the multimorbidity and cognitive decline which characterizes many of the patients.

A comparison with similar studies from hospital CECs is illuminating (Kalager et al., 2011; Magelssen et al., 2020b; Reiter-Theil & Schürmann, 2016). The large categories are similar, with some notable differences: On the one hand, issues about decision-making and care at the end of life were less prominent in our study. End-of-life care is of course central to nursing homes and is common also in home care. A potential explanation of the relative lack of cases about this issue could be that the services have given much attention to this field in recent years and that helpful guidelines exist (e.g., Norwegian Directorate of Health, 2013). Professionals might have considered themselves competent to handle issues without ethics support. There were also fewer cases about information and communication than in hospitals. One reason could be that complex medical diagnostics and start-up of medical treatment less often take place in the primary care services that have used the CECs.

On the other hand, two categories apparently more specific to the primary care sector were prominent in our material. Issues where the interests of the one go against the interests of the many arise in services where patients live together, such as nursing homes or sheltered housing. Here, interests may conflict more directly than in hospitals. Professionalism was another significant category. Although issues of professionalism are bound to arise also in hospitals, the primary care setting is characteristic in that many professionals work alone, and/or in patients’ homes. This may lead to specific challenges (Heggestad et al., 2020).

### Off to a Good Start

CEC work is complex and requires skill, dedication and time (ASBH, 2011; Magelssen et al., 2020a; Schildman et al., 2016). In this light, 2.5 years is not a long time and expectations of what newly established CECs can accomplish in that time should be tempered (Lillemoen et al., 2016). In several respects, the CECs have exceeded our expectations. Most of all, they have received and discussed a large number of cases. This goes especially for two of the CECs. In a study of the well-established CEC system in Norwegian hospitals, CECs reported discussing 4.7 cases yearly on average (Magelssen et al., 2018). Remarkably, our four CECs all exceeded that level in their first years of operation. They have also begun the outreach to professionals and managers in the municipality.

The present study was not designed to elucidate the factors that have been most instrumental in promoting implementation, but hypotheses can be formulated. We deem it likely that three factors have been significant: the CME’s training package and implementation support; the expectations and obligations incurred for the municipality and the resource persons in committing to participating in the research project; and the professional networks and personal dedication of the resource persons.

### Several Services and Professions are Absent

However, although the CECs have received many cases, the distribution of services and professions who have used the CECs is severely skewed. We see three potential explanations. First, in a Norwegian context, healthcare institutions—hospitals and nursing homes—have traditionally been most active in putting ethics on the agenda. Second, the CME which has been responsible for training CEC resource persons and members is originally a centre for *medical* ethics, and this is reflected in the case examples and emphasis in the training. The CECs might have “inherited” this bias towards healthcare and might have shown lesser interest in other services. Third, in the CME’s experience, GPs have been a particularly difficult group to reach with systematic ethics work and training. Partly this might be due to how the GPs are particularly pressed for time and remunerated for activities, thus lacking incentives to participate in ethics deliberations. Yet, they might be important to include in discussions concerning patients for whom they are responsible, and so inviting the GP should always be considered. Their participation might be very relevant for instance for patients receiving home-based care, where responsibility is shared between the GP and the home care services.

In our view, the CECs’ approach to case deliberations is a suitable model also for services other than nursing homes and home care (see, e.g., Heggestad et al., 2020). We see no reason to think that ethical problems arise less often in these other services. The CECs could prioritize outreach to these services, thus ensuring that the services are informed about what the CEC can offer and lowering thresholds for inquiries.

We were surprised to see that mid-level managers used the CEC more often than staff directly involved in patient care. One reason might be that this group has received more information about the CEC. Another interpretation is that managers contact the CEC *on behalf of* their employees. The finding also fits with the impression that mid-level managers in Norwegian municipal care have particularly central roles in handling difficult problems that arise in the services. They have responsibility both for their staff and for quality and routines. Both staff and top-level managers might expect them to handle and decide on challenges and value conflicts. Considering these expectations and responsibilities the CEC might be a particularly welcome support for this group (Magelssen & Karlsen, 2021).

### The Relative Absence of Next of Kin and Patients

Although staff were present in 40 of 54 case deliberations, next of kin only attended 10 deliberations, and patients were never present. Only the two CECs with the highest number of deliberations had had next of kin present. We consider it good practice to conduct case deliberations with next of kin present and recommend that at the very least it is always considered seriously whether patients or next of kin ought to be invited. When the problem is staff’s reluctance to involve next of kin, for instance because of concerns that it will not be possible to speak freely, the CEC should address this. Perhaps next of kin should sometimes be invited to the CEC by the CEC leader and not the health professional. The presence of next of kin can be important because their views and knowledge of the patient are significant, because the case deliberation can contribute to conflict resolution, and to signal that they are taken seriously (Førde & Linja, 2015; Magelssen et al.,  2020b). Many cases concerned patients with reduced decision-making capacity, so their presence in the discussions is perhaps often not advisable. Still, in the age of patient autonomy and empowerment the absolute absence of patients and the relative absence of next of kin might make the CECs reconsider their policies on including stakeholders. The absence of relevant stakeholders introduces a risk of bias (Magelssen et al., 2014). However, in this study, we have no way of assessing independently whether patients or next of kin *ought* to have been invited in a given case.

It is also notable that all cases discussed in the CECs originated from employees of the services; not a single case was initiated by patients or next of kin. This corresponds with our experience from the hospital CECs. Yet it is not the CECs’ intention to be a forum only for staff. We consider it likely that there have been many moral problems experienced as such primarily by service users and their relatives (e.g., insufficient services and neglect of patient needs) that have thus not reached the CECs.

### Strengths and Limitations

Two of the authors have been central in the project from the outset, in training and follow-up of the CECs, and so are not fully independent as evaluators of the CECs’ work. Author LT, on the other hand, has not been thus involved. The present study is a relevant evaluation of some aspects of the CECs’ work, but has to a large extent consisted in collecting quantitative data about the CECs. We have not evaluated the *quality* of the case reports and deliberations. Thus, in itself this investigation is unable to judge the four new CECs as “successful” or not.

## Conclusion

The investigation of the structure and activities of new CECs in four Norwegian municipalities during their first 2.5 years of operation indicates that it is feasible to attain a high level of activity including case deliberations within the time frame. Most of all, however, it confirms that significant, characteristic, difficult and complex moral problems arise in primary care services. Evaluations of whether a CEC is a feasible and suitable means of ethics support for primary care services should be supplemented also with other methods. In particular, there is a need to explore how patients and next of kin can be included more often, and to examine whether and to what extent CEC deliberations have an impact on practice.
